# Rab7 inhibitor enhances stem cell differentiation into keratinocyte-like cells with anti-inflammatory properties

**DOI:** 10.3389/fimmu.2025.1503007

**Published:** 2025-05-26

**Authors:** Raghad Alghazali, Mouna Tabebi, Moustafa Elmasry, Ahmed El-Serafi

**Affiliations:** ^1^ The Department of Biomedical and Clinical Sciences (BKV), Linköping University, Linköping, Sweden; ^2^ Clinical Genomics Linköping, Linköping University, Linköping, Sweden; ^3^ Department of Hand Surgery and Plastic Surgery and Burns, University Hospital, Linköping, Sweden

**Keywords:** regenerative medicine, wound healing, stem Cells, CID-1067700, Rab7, differentiation, Ras pathway, skin

## Abstract

Management of difficult-to-heal skin wounds presents a significant clinical challenge, particularly when associated with inflammatory skin disorders. The differentiation of stem cells into keratinocyte-like cells has been explored as a potential regenerative therapy. Ras-related protein (Rab) 7, a key regulator of membrane trafficking, influences the activity of several growth factors. In this study, the competitive Rab7 inhibitor, CID-1067700, was investigated for the differentiation of adipose-derived stem cell into keratinocyte-like cells. This treatment upregulated several epidermal markers, including P63, cytokeratin 5 and 14 and filaggrin, while downregulated the stem cell marker, vimentin. Microarray data showed upregulation of the anti-inflammatory gene HMOX-1, coupled with the downregulation of various inflammation-related pathways, such as TNF, chemokine, AGE-RAGE and IL-17 signalling cascades, as well as cytokine-cytokine receptor interaction pathways. Gene set enrichment analysis predicted Ehmt2, an epigenetic regulator, to be the top activated upstream regulator, enhancing the transcriptional activity. Protein array analysis showed reduced secretion of several inflammatory cytokines, including IL-1α, IL-8, IL-17A, and IL-32, while enhancing the secretion of the epidermal growth factor. Our findings provide preliminary evidence that CID-1067700, as an additive to the differentiation media, not only enhances stem cell differentiation into keratinocyte-like cells, but also improves their anti-inflammatory properties. These combined regenerative and anti-inflammatory properties may offer significant therapeutic potential for patients with chronic skin wounds, particularly those with underlying inflammatory conditions.

## 1 Introduction

The skin functions as a multi-layered barrier against environmental hazards and plays a crucial role in various physiological processes, including thermoregulation and vitamin D synthesis. Despite its remarkable regenerative capacity, the skin remains vulnerable to injury and disease, with chronic skin wounds representing a significant clinical challenge. These wounds deviate from the normal healing process, persist in the inflammatory phase and fail to heal within the typical timeframe of three months (1). The impaired healing observed in chronic wounds is primarily attributed to dysregulated inflammatory responses, characterized by an imbalance between pro- inflammatory and anti-inflammatory cytokines (2). Furthermore, elevated levels of reactive oxygen species (3) and proteases (4) exacerbate cellular damage and degrade extracellular matrix, thereby further impeding the wound healing process.

Chronic wounds are frequently associated with an underlying condition, such as diabetes, venous insufficiency, and arterial insufficiency (5, 6). Notably, over 20% of chronic wounds exhibit inflammatory or autoimmune components, complicating wound management and significantly increasing patient morbidity. This leads to doubling of the failure rate for standard wound management procedures (7). Systemic autoimmune diseases are frequently associated with lower leg ulcers, which further challenges the therapeutic interventions and prolongs the healing process. For example, rheumatoid arthritis is linked with leg ulcers in more than 26% of the patients on long-term follow-up (8). Similarly, scleroderma is associated with lower leg ulcers in 4% of patients, with 70% presented bilaterally (9). In cases of pyoderma gangrenosum, surgical intervention can exacerbate skin wounds associated with ruptured bullae (7). The classical treatment for these conditions is long-term corticosteroids, which are known to impair skin wound healing (10).

In developed nations, the prevalence of chronic wounds ranges from 1% to 2% of the general population, with incidence rates exceeding 5% among the elderly (11). The economic burden of chronic wounds is substantial, with healthcare expenditures exceeding €16.8 billion in Germany, SEK 23 billion in Sweden, £8.8 billion in the UK, and $145 billion in US (12–14). Moreover, these wounds profoundly impact the patients’ quality of life, causing chronic pain, depression, anxiety, and social isolation (1).

Traditional treatments for chronic wounds, such as skin grafts, often prove insufficient and may result in complications at the donor site (15). The advancements in biomaterial production have led the development of novel wound dressings which can offer help for some patients. Unfortunately, there are challenges associated with these dressings, including incorporation in the wound, hypersensitivity to animal-derived components, cost and availability (16). Stem cell-based therapy has emerged as a promising approach in wound repair, offering anti-inflammatory properties and an ability to accelerate the healing process (17). Adipose-derived stem cells (ASCs) demonstrate significant potential for treating inflammatory and autoimmune skin conditions due to their anti-inflammatory paracrine secretion and regenerative capabilities (18). The therapeutic potential of ASCs in managing chronic wounds is particularly noteworthy. Studies have shown ASCs ability to transdifferentiate into various cell lineages, including keratinocyte-like cells (KLCs) *in vitro* (19–21). Cellular differentiation is known to induce dynamic changes in gene expression, protein secretion, and physical properties, including membrane trafficking. The latter has emerged as a critical regulator governing stem cell differentiation and fate acquisition, mediated through pivotal signalling pathways (22). Ras-related protein 7 (Rab7) is a master regulator of membrane trafficking and plays essential roles in endosome maturation, vesicle transport from early to late endosome, lysosome biogenesis, and autophagy via its GTPase activity (23, 24). Thus, Rab7 influences the activity, localization, and degradation of signalling receptors and ligands involved in key developmental pathways, including fibroblast growth factor (25), epidermal growth factor (26), and ligands from the Wnt (27), Notch (28) and Hedgehog (29) pathways. Interestingly, Rab7 influences various signalling pathways that are activated during inflammation. For instance, it can affect the NF-κB pathway, which is a key regulator of inflammatory responses (30).

Our unpublished data identified Rab GDP dissociation inhibitor alpha as a key secretion by keratinocytes that could be responsible for enhancing the differentiation of ASCs into KLCs. As Rab7 is a known target for this inhibitor, we hypothesized that inhibiting Rab7 with a small molecule could enhance the differentiation of ASCs into KLCs. Thus, this study aimed to investigate the molecular mechanisms underlying Rab7-mediated stem cell differentiation into keratinocyte-like cells, through comprehensive molecular analyses as well as protein profiling. We hypothesized that Rab7 inhibition enhances ASC differentiation into KLCs with anti-inflammatory properties via altered membrane trafficking.

## 2 Materials and methods

### 2.1 Cell culture

The adipose-derived mesenchymal stem cell (ASCs) line, ASC52telo (SCRC-4000, ATCC) immortalized with human telomerase reverse transcriptase (hTERT), were selected for their consistent stem cell properties and reproducibility. This cell line is widely used in literature for modelling primary stem cells and showed comparable findings to primary cells in terms of biology, metabolism, differentiation and even gene expression pattern (21, 31–33). ASCs were expanded in Dulbecco’s Modified Eagle Medium (DMEM; Thermo Fisher, Cat. 31885049) supplemented with 10% fetal bovine serum (FBS, Thermo Fisher, Cat. 10500064) and 1% penicillin/streptomycin, in a humidified incubator at 37°C with 5% CO₂.

### 2.2 Cell viability assay

The cytotoxicity of CID-106770 (Sigma-Aldrich, Cat. SML0545) was examined using the 3-(4,5-Dimethylthiazol-2-yl)-2,5-Diphenyltetrazolium Bromide (MTT) assay. Briefly, 3 × 10^3^ cells in 200 μl of DMEM were seeded onto 96-well culture plates and the concentrations of 0, 20, 40, 60, 80, and 100 μM of CID-106770 were applied for 24 or 72 hours. A vehicle control of cells treated with 0.1% DMSO (Sigma-Aldrich, Cat. 472301) were also included in the experiment. The MTT solution (5 mg/ml; Sigma-Aldrich, Cat No. M5655) was added to each well, and the cells were incubated at 37°C for 4 hours. The formazan crystals were dissolved in 200 μl DMSO, and the absorbance was measured at 570 nm (Spectra Max plus 384, Molecular Devices). The normalized absorbance of each treatment was divided by the absorbance of untreated cells to calculate the fold change of cell survival relative to untreated control.

### 2.3 Cell differentiation and treatment

**Figure 1:**
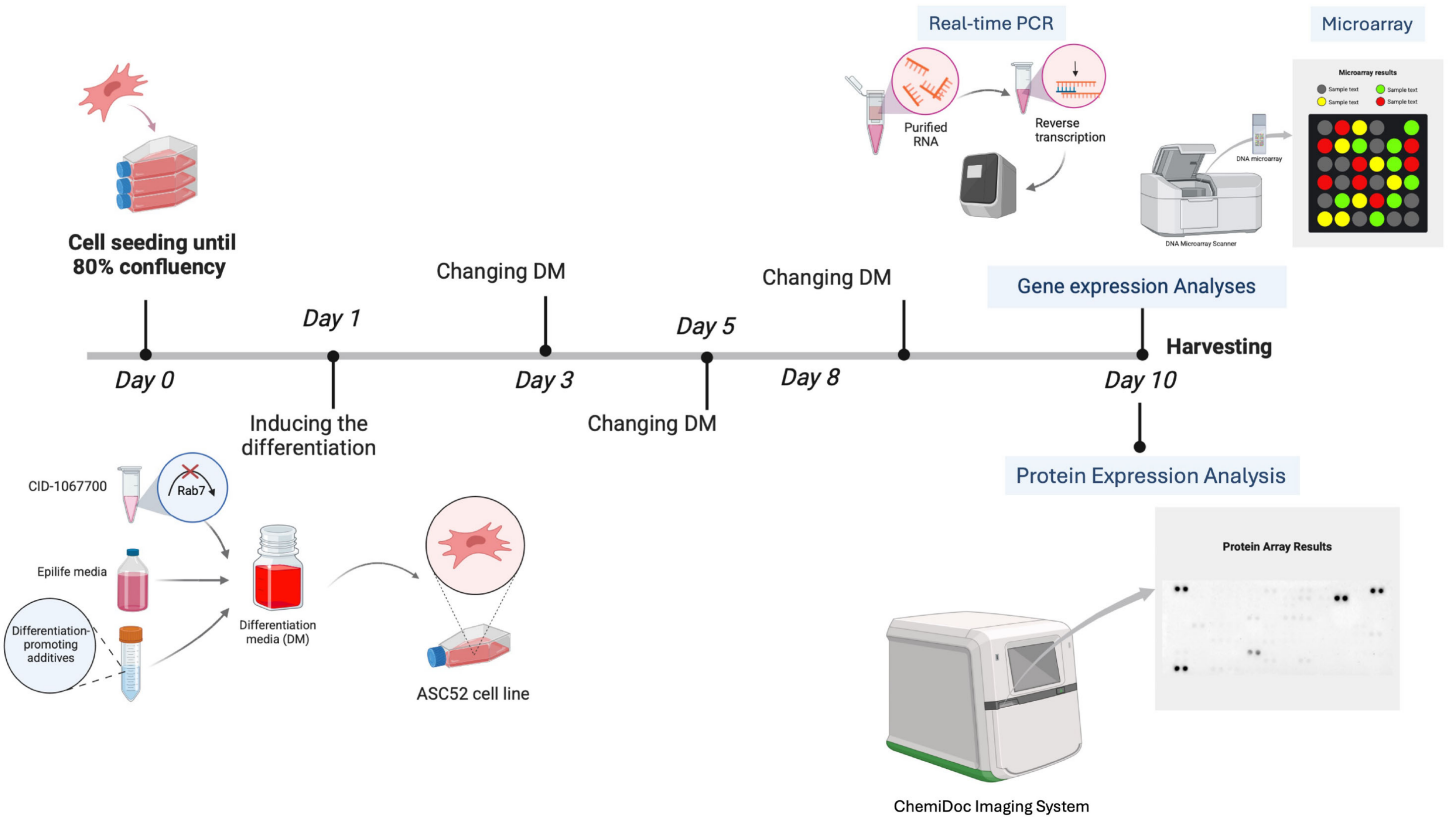
A simplified illustration of the experimental design. Figure created with BioRender.com.

To induce the differentiation of ASCs into KLCs, the cells were cultured in a differentiation media (DM) consisted of EpiLife™ Medium (ThermoFisher, Cat. MEPI500CA) supplemented with EpiLife™ Defined Growth Supplement (EDGS, ThermoFisher, Cat. S0125) and 1% penicillin/streptomycin, along with previously published differentiation-inducing additives summarized in **Table 1** (34). The cells were cultured on 2 μg/cm² of collagen type I from rat tail (Fisher Scientific, Cat. 10114532). The study included three experimental groups: a control group with cells cultured in DM, a vehicle control group with cells treated with DM and 0.1% DMSO, and a treatment group that received DM and Rab7 inhibitor, CID-1067700 at 40 μM. The differentiation media, along with the respective additives, was changed twice weekly, for ten days (**Figure 1**).

**Table 1:** The differentiation-promoting additives used in this study.

Component	Concentration	Provider/Cat. number
Human Recombinant BMP-4 (BMP-4)	0.5 nM	STEMCELL Technologies, Cat. 78211
Human recombinant epidermal growth factor (EGF)	3.2 nM	STEMCELL Technologies, Cat. 78006.1
Ascorbic acid 2-phosphate (Asc-2-P)	0.3 mM	Sigma-Aldrich, Cat. A5960
Hydrocortisone (HC)	1.38 µM	Sigma-Aldrich, Cat. H4001
All-trans retinoic acid (ATRA)	5 nM	Sigma-Aldrich, Cat. R2625

### 2.4 Gene expression analysis

#### 2.4.1 RNA extraction

Total RNA was extracted using the RNeasy Plus Mini kit (Qiagen, Cat. 74134), according to the manufacturer’s instructions. RNA was quantified using a Nanodrop spectrophotometer (ThermoFisher, Cat. 701-058112) and reverse transcribed with the Maxima™ First Strand cDNA Synthesis Kit (ThermoFisher, Cat. K1642).

#### 2.4.2 Microarray analysis

RNA samples were assessed for size and integrity using the Agilent 2100 Bioanalyzer (Agilent Technologies). Samples with RNA Integrity Number (RIN) ≥ 9.6 were hybridized with the Human Clariom™ S microarray kit (Thermo Fisher Scientific, Cat. 902926), following the manufacturer’s recommended protocol. Post-hybridization, the arrays were washed and stained using the GeneChip™ Expression Wash, Stain, and Scan Kit (Thermo Fisher Scientific, Cat. 900720) and the GeneChiptm Fluidics Station 450 (Thermo Fisher Scientific) to remove non-specific binding and enhance signal detection. Subsequently, the arrays were scanned with the GeneChip Scanner 3000 7G (Affymetrix, Santa Clara). Data pre-processing, quality control, and the calculation of log2 fold-changes between sample expression values were performed using the Transcriptome Analysis Console Software (Affymetrix). Differentially expressed genes (DEGs) were identified based on a log2 fold change value |FC| of ≥ 1.5 and an adjusted false discovery rate (FDR) p-value < 0.05.

#### 2.4.3 Bioinformatic analysis

Pathway enrichment analysis was conducted on all statistically significant DEGs using iPathwayGuide™ (Advaita Bioinformatics, MI, USA). The gene interactions and pathways were computed with reference to the Kyoto Encyclopaedia of Genes and Genomes (KEGG) database (www.genome.jp/kegg). In addition to pathway enrichment analysis, the most significantly enriched gene ontology (GO) biological processes associated with DEGs were evaluated using the iPathwayGuide software. Only differentially expressed genes exhibiting a fold change of ≥ |1.5| and a false discovery rate (FDR) of ≤0.05 were included in the analysis.

#### 2.4.4 Real-time Polymerase chain reaction

Real-time polymerase chain reaction (qPCR) was performed using the 7500 Real-Time PCR System (Applied Biosystems, Cat. 4351104) and the PowerUp™ SYBR™ Green Master Mix (Fisher Scientific, Cat. 15390929), following the manufacturer’s instructions. The primer sequences are listed in **Table 2**. The following thermal cycling conditions were used: UDG (uracil-DNA glycosylase) activation at 50°C for 2 minutes, polymerase activation at 95°C for 2 minutes, followed by 40 cycles of denaturation at 95°C for 3 seconds and annealing/extension at 60°C for 30 seconds. Gene expression levels were normalized to *GAPDH* and calculated relative to the control group using the 2^-∆∆Cq^ method.

**Table 2:** List of primer sequences used in this study.

Gene	Accession number	Fwd Primer (5´➔3´)	Tm	Rev Primer (3´➔5´)	Tm
*ITGB8*	NM_002214	CAGCACTGTGTCAATTCAAAGG	62.5	GCAGGCTGTATAACAGGTGGG	65.1
*MGP*	NM_000900	TTTGTGTTATGAATCACATGAAAGC	61.3	GAGCGTTCTCGGATCCTCT	63.1
*HMOX-1*	NM_002133	GTGCCACCAAGTTCAAGCAG	64.2	GCAACTCCTCAAAGAGCTGGA	64.7
*COL1A1*	NM_000088	GCCAAGACGAAGACATCCCA	64.5	CCACACGTCTCGGTCATGG	64.7
*COL8A1*	NM_001850	ATCACAGCCCTTCCCCGAT	65.6	AACGTGTGAGCTCCCTTGAG	64.4
*FGF7*	NM_002009	AGGGACCCAAGAGATGAAG	60.7	TGATTGCCACAATTCCA	56.3
*VCAM1*	NM_080682	GTCTCCAATCTGAGCAGCAA	62.4	TGGGAAAAACAGAAAAGAGGTG	61.5
*CK5*	NM_000424.4	CAGAGCCACCTTCTGCGTCCTG	69.3	GCTGAAGCTACGACTGCCC	65.0
*CK14*	NM_000526.5	CCTCCTCCAGCCGCCAAATCC	69.6	TTGGTGCGAAGGACCTGCTCG	69.2
*Filaggrin*	NM_002016.2	TGAAGCCTATGACACCACTGA	63.4	TCCCCTACGCTTTCTTGTCCT	65.4
*Vimentin*	NM_003380.5	AAGACACTATTGGCCGCCTG	64.8	GGCAGAGAAATCCTGCTCTC	62.5
*GAPDH*	NM_001357943.2	CCTGCACCACCAACTGCTTA	65.0	GGCCATCCACAGTCTTCTGAG	64.7

### 2.5 Proteome profiling

Cell culture supernatant was collected on day 10 of differentiation. The differential protein expression between the control and treatment group was assessed using the Proteome Profiler™ Human XL Cytokine Array (R&D Systems, Cat. ARY022B), according to the manufacturer’s protocol. Briefly, the array membranes were incubated with the blocking buffer for one hour on a rocking platform, followed by overnight incubation at 4°C with 500 μL of cell culture supernatant. The arrays were then incubated with a cocktail of biotinylated detection antibodies for one hour, followed by chemiluminescent detection using Streptavidin-HRP. The membranes were imaged using the ChemiDoc™ MP imaging system (Bio-Rad, Cat. 12003154), and the pixel intensities were analysed using Fiji (35).

### 2.6 Surface protein characterization

Immunofluorescence staining was conducted to assess the differentiation of ASCs into KLCs based on their expression of epithelial differentiation markers. The cells were fixed with 4% ice-cold methanol for 20 minutes and permeabilized using 0.5% Triton X-100 for 15 minutes. Nonspecific binding was blocked using 3% bovine serum albumin (Sigma-Aldrich, Cat. A9418). The cells were incubated with the primary antibodies (listed in **Table 3**) overnight at 4°C and with the fluorescently labelled secondary antibodies for one hour at room temperature. Nuclear counterstaining was performed using NucBlue™ Fixed Cell ReadyProbes™ Reagent (DAPI, Fisher Scientific, Cat. R37606) for 2 minutes, followed by rinsing with PBS. The cells were visualized at 20x magnification using a fluorescent inverted microscope (DMi8, Leica), and image analysis was conducted using Fiji (35) by dividing the fluorescence intensity of positive cells by the total number of cells. Data are reported as a fold change in fluorescence intensity unit per cell (FU/cell) and relative to the cells treated with differentiation media only.

**Table 3:** List of the antibodies used for the immunofluorescence staining.

Antibody	Dilution factor	Company/Cat. number
Rabbit anti-Cytokeratin 5 [EP1601Y]	1:100	Abcam, ab52635
Mouse anti-Cytokeratin 14 [LL002]	1:500	Abcam, ab7800
Rabbit Anti-Filaggrin antibody	1:500	Abcam, ab81468
Goat Anti Rabbit IgG H&L (Alexa Fluor 488)	1:500	Abcam, ab150077
Goat anti Mouse IgG (H+L) Secondary Antibody (Alexa Fluor 647)	1:1000	Invitrogen, A-21235
Goat anti-Rabbit IgG (H+L) Cross-Adsorbed Secondary Antibody (FITC)	1:50	Invitrogen, F2765

### 2.7 Statistical analysis

The data are presented as mean ± standard deviation (SD) of at least three experimental replicates, unless stated otherwise. Statistical analysis was conducted using IBM SPSS Statistics software, version 29. Statistical differences were determined by independent samples t-test or one-way analysis of variance (ANOVA), followed by Tukey’s *post hoc* test for cases with equal variance or Dunnett’s T3 test for cases with unequal variance. The homogeneity of variances was confirmed using Levene’s Test. p-value < 0.05 was considered statistically significant.

## 3 Results

### 3.1 CID-106770 did not affect the cell viability at the studied concentrations

**Figure 2:**
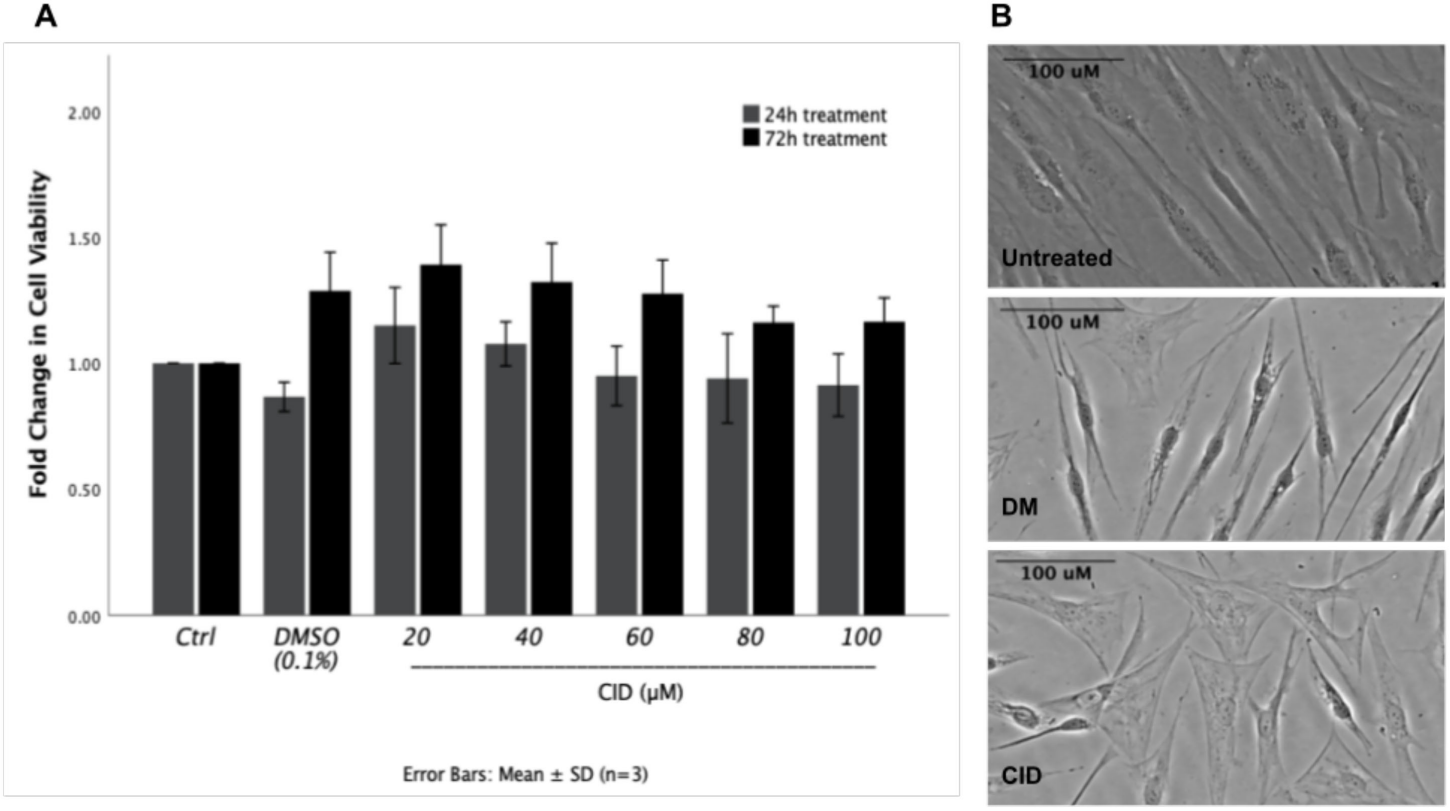
Effect of Rab7 inhibition on the viability and morphology of ASCs. **(A)** Viability of ASCs after treatment with different concentrations of CID-1067700 at 24 and 72 hours. No significant changes in cell viability were observed. **(B)** Representative bright-field images illustrating the morphological changes in ASCs after 10 days of treatment with differentiation media (DM) with and without 40 μM CID-1067700, in comparison to undifferentiated ASCs. Scale bars = 100 μm.

The potential cytotoxic effect of CID-106770 on ASCs viability was evaluated after 24-hour and 72-hour. None of the tested concentrations (20, 40, 60, 80, and 100 μM) of CID-106770 or the vehicle control (0.1% DMSO) exhibited a significant cytotoxic effect on ASCs cell viability when compared to untreated cells (**Figure 2A**). CID-1067700 was selected at 40 μM for further experiments as this concentration was demonstrated in literature to efficiently inhibit Rab7 activity, while maintaining cell viability and minimizing cytotoxicity (36–38).

### 3.2 ASCs change of morphology upon treatment

Morphological changes were observed in ASCs after 10 days of treatment with differentiation media supplemented with CID-1067700. Untreated ASCs displayed typical fibroblast-like morphological characteristics. However, after 10 days of differentiation, cells treated with CID-1067700 showed high number of flattened cells (**Figure 2B**).

### 3.3 Rab7 inhibitor induced transcriptomic changes in ASCs

**Figure 3:**
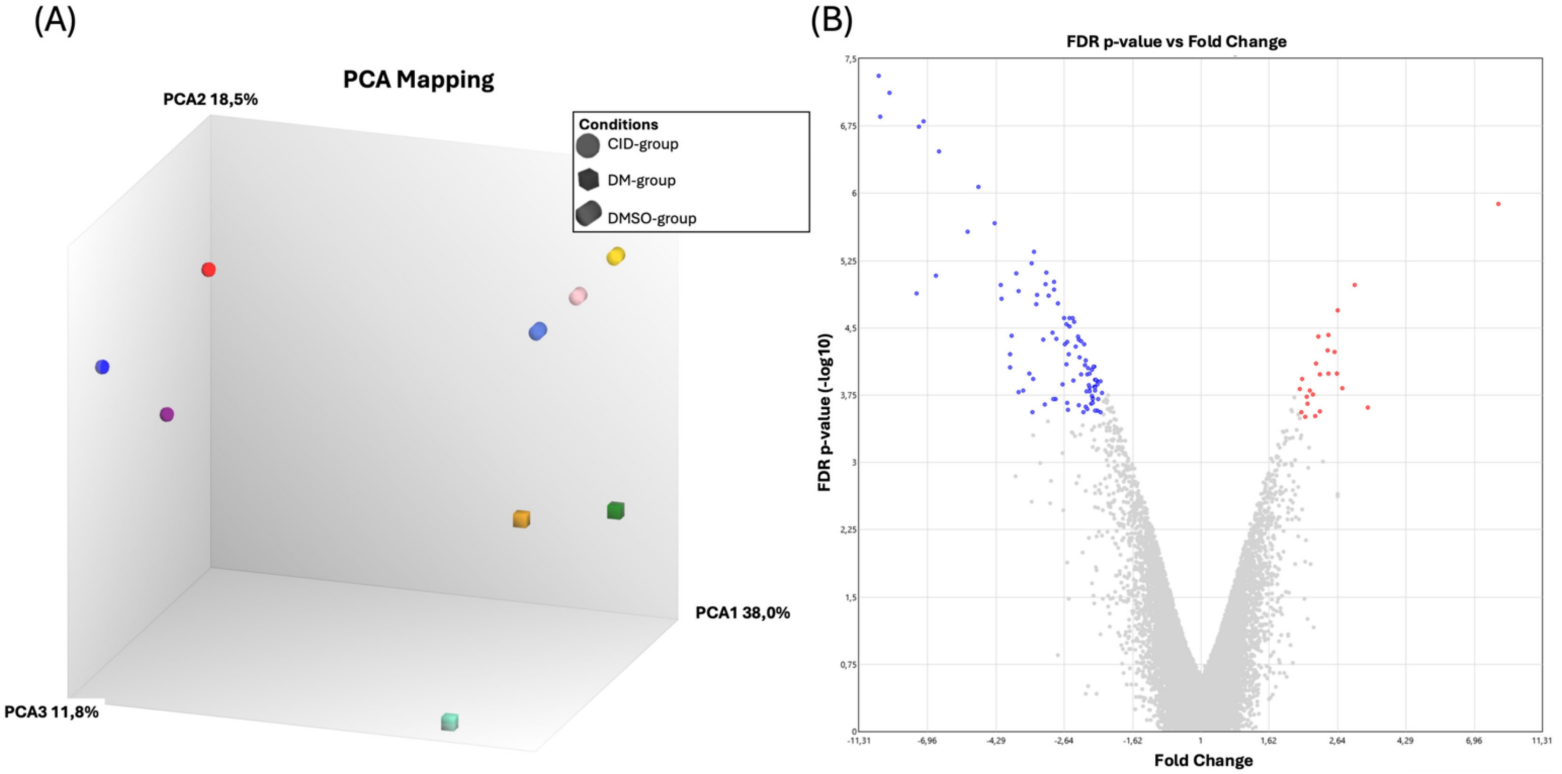
Rab7 inhibition leads to transcriptomic changes in ASCs. **(A)** Principal component analysis (PCA) of the transcriptomic profiles of cells treated as control (DM), with vehicle (DMSO), or with CID. PCA revealed clustering of the vehicle group with DM group, indicating a high degree of similarity between these two groups. **(B)** Volcano plot showed the differentially expressed genes (DEGs) in the CID-treated ASCs compared to those treated with DM. Downregulated genes are shown in blue, upregulated genes are in red, while insignificantly regulated genes are in grey.

Microarray gene expression profiling was conducted to elucidate the genes and regulatory pathways influenced by Rab7 inhibition in ASCs. Principal Component Analysis (PCA) (**Figure 3A**) and Heatmap analysis (**Supplementary Figure S1**) of the transcriptomic profiles revealed that the vehicle group (DMSO) and the differentiation media control group (DM) were clustered together, indicating a negligible effect of the vehicle. Therefore, subsequent analyses focused on comparing the differences between CID-treated ASCs and those treated with DM. Out of 21,448 studied genes,134 DEGs were identified in ASCs treated with CID versus DM (**Supplementary Table 1**). Among these DEGs, 107 were downregulated and 27 were upregulated (**Figure 3B**).

### 3.4 Rab7 inhibition altered key signalling pathways and biological processes in ASCs

**Figure 4:**
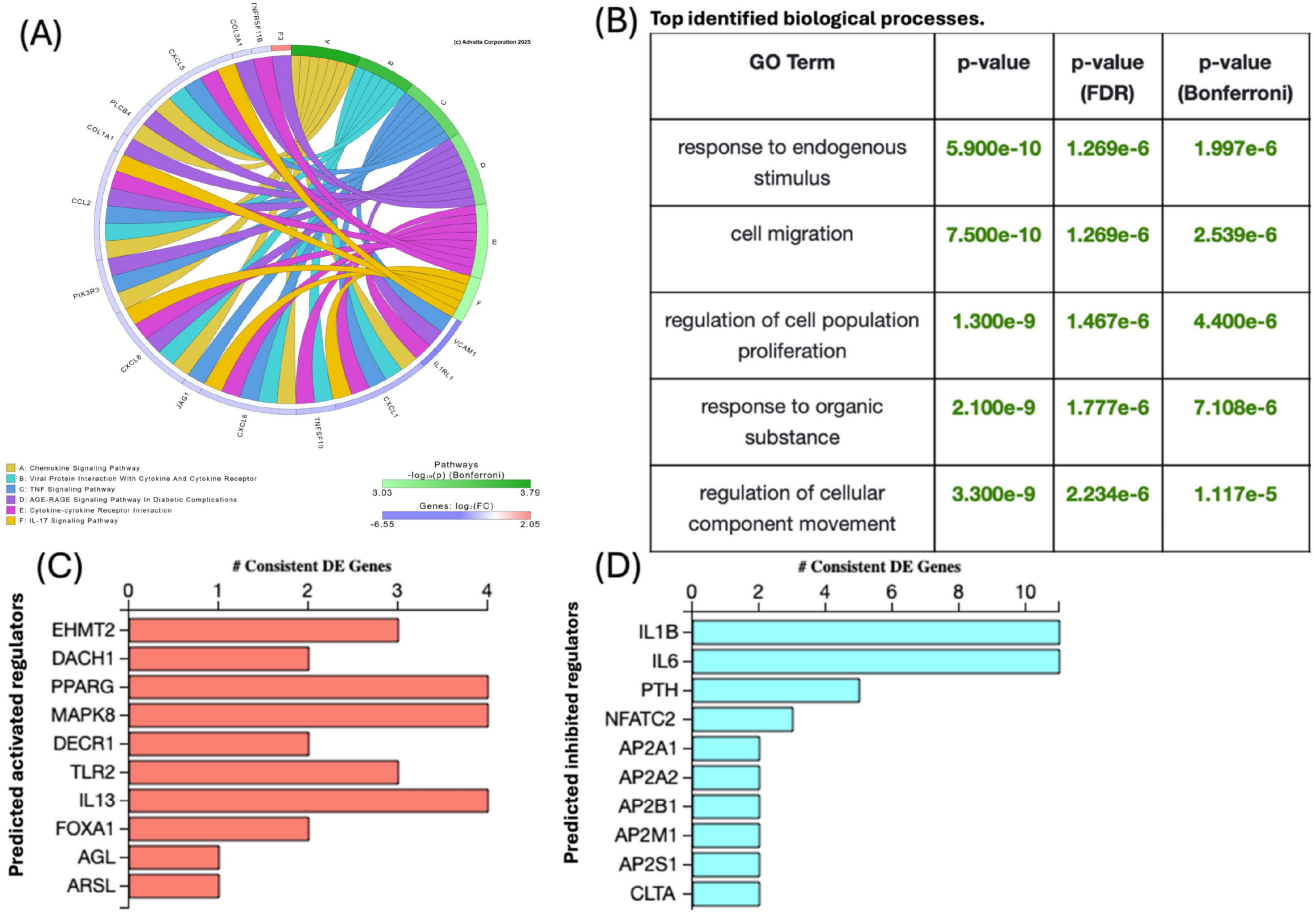
Impact of Rab7 inhibition on signalling pathways, biological processes, and upstream regulators in ASCs. **(A)** Chord Diagram of the top six significantly enriched pathways. **(B)** The top five enriched GO biological processes among the DEGs in the CID-treated ASCs. **(C)** Predicted activated or **(D)** inhibited upstream regulators for the DEGs in CID-treated ASCs compared to the control group. These predictions were made using the iPathwayGuide software.

The identified DEGs were used as input for the iPathwayGuide software to determine significantly impacted pathways in response to CID-treatment. A total of 92 pathways were affected by CID treatment, encompassing: *Chemokine Signalling Pathway* with key genes such as *VCAM1, CXCL1*, and *CXCL8* showing differential expression. This pathway is crucial for regulating immune cell migration and inflammatory responses. The *Viral Protein Interaction with Cytokine and Cytokine Receptor Pathway* also showed significant changes, with *PIK3R3*, *CCL2*, and *COL1A1* genes being differentially expressed, indicating potential implications in immune response modulation and viral pathogenesis. Additionally, the *TNF Signalling Pathway* was notably impacted, with key genes including *TNFSF10, CXCL6, and JAG1*. This suggests a possible role in inflammation and apoptosis. The *AGE-RAGE Signalling Pathway* in Diabetic Complications was another critical pathway affected, with genes such as *CXCL5, COL3A1*, and *TNFRSF11B* showing differential expression, highlighting its importance in diabetic complications and cellular stress responses. The *Cytokine-Cytokine Receptor Interaction Pathway* was significantly affected, with *IL1RL1, CXCL8*, and *PIK3R3* genes being key differentially expressed genes, underscoring its role in immune signalling and inflammation. Lastly, the *IL-17 Signalling Pathway* was significantly impacted, with *CXCL1, CXCL6*, and *JAG1* genes showing differential expression, indicating its role in inflammatory and autoimmune responses (**Figure 4A**).

We identified the top five biological processes significantly impacted by the differentially expressed genes in CID treated cells. These include the response to endogenous stimulus, cell migration, regulation of cell population proliferation, response to organic substance, and regulation of cellular (**Figure 4B**).

In this study, we identified several upstream regulators that are predicted to be either activated or inhibited, playing crucial roles in the differential expression of genes in CID treated cells. Among the activated regulators, EHMT2 (euchromatic histone lysine methyltransferase 2) stands out as a significant regulator. The differential expression of EHMT2 was consistent with its predicted activation, affecting key genes such as *VCAM1*, *PDK4*, and *CXCL8*. Other notable upstream regulators predicted to be activated include DACH1, PPARG, MAPK8, DECR1, TLR2, IL13, FOXA1, AGL, and ARSL. For instance, PPARG (peroxisome proliferator-activated receptor gamma) is involved in the regulation of adipogenesis and inflammation, while TLR2 (toll-like receptor 2) plays a critical role in the immune response to microbial infections. IL13 (interleukin 13) is a cytokine involved in the regulation of inflammatory responses, and FOXA1 is a transcription factor involved in gene expression during development (**Figure 4C**).

Conversely, several upstream regulators were predicted to be inhibited, including IL1B (interleukin 1 beta) and IL6 (interleukin 6), both key cytokines involved in the regulation of inflammatory responses and immune modulation (i.e., chronic inflammation and wound healing). Their predicted inhibition suggests a potential downregulation of inflammatory pathways, which could have implications for immune response and disease progression. Other inhibited regulators include PTH (parathyroid hormone), NFATC2 (nuclear factor of activated T-cells, cytoplasmic 2), and several adaptor proteins such as AP2A1, AP2A2, AP2B1, AP2M1, AP2S1, and CLTA. The inhibition of PTH suggests potential effects on calcium homeostasis and bone metabolism, while the inhibition of NFATC2 indicates possible impacts on T-cell activation and immune function (**Figure 4D**).

### 3.5 Differential gene expression was consistent by qPCR

**Figure 5:**
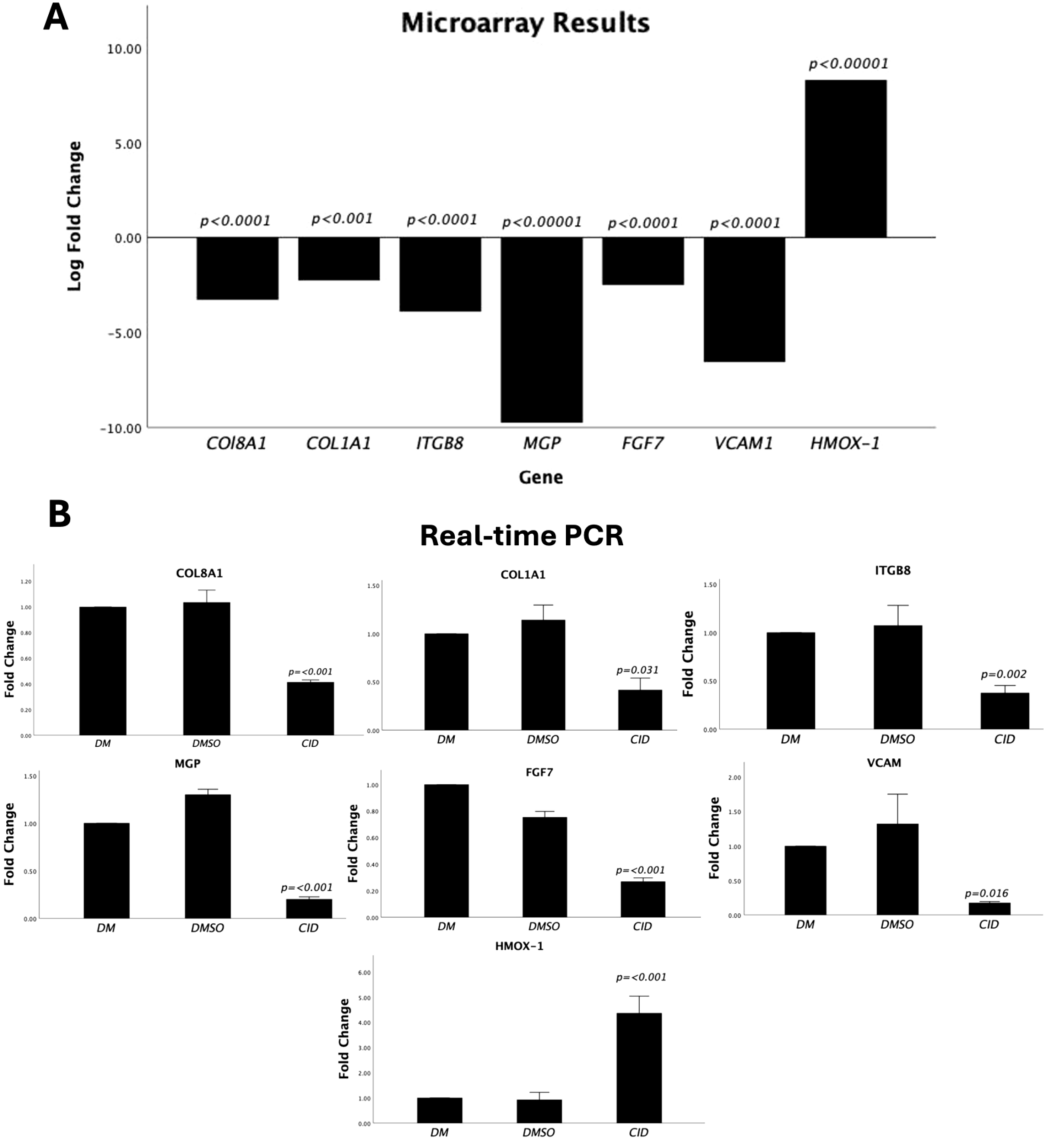
Validation of the expression levels of the differentially expressed genes identified by microarray with qPCR. **(A)** Bar chart displaying the relative expression levels of seven selected DEGs (*COL81A, COL1A1, ITGB8, MGP, FGF7, VCAM1, and HMOX-1)*. Bars represent the log2-fold changes in gene expression in CID-1067700-treated ASCs **(CID)** compared to the control group (DM). **(B)** Validation of the expression levels of the selected genes by qPCR. The expression levels are normalized to *GAPDH* and calculated relative to the expression of ASCs treated with DM only using the 2^-∆∆Cq^ method. Data are presented as mean ± standard deviation (SD) of three experimental replicates. The vehicle control was added to the qPCR to validate similarity with the negative control.

To ensure the reliability of the microarray analysis, the expression levels of seven DEGs identified by the microarray analysis *(COL81A, COL1A1, ITGB8, MGP, FGF7, VCAM1, and HMOX-1)* were validated using qPCR. These genes were selected based on their relevance to keratinocyte functions or to validate their potential significance in ASCs differentiation. A strong correspondence between the microarray (**Figure 5A**) and the qPCR results (**Figure 5B**) was observed for all selected genes, confirming the reliability and accuracy of the microarray findings.

### 3.6 Rab7 inhibition induced proteomic changes in ASCs

**Figure 6:**
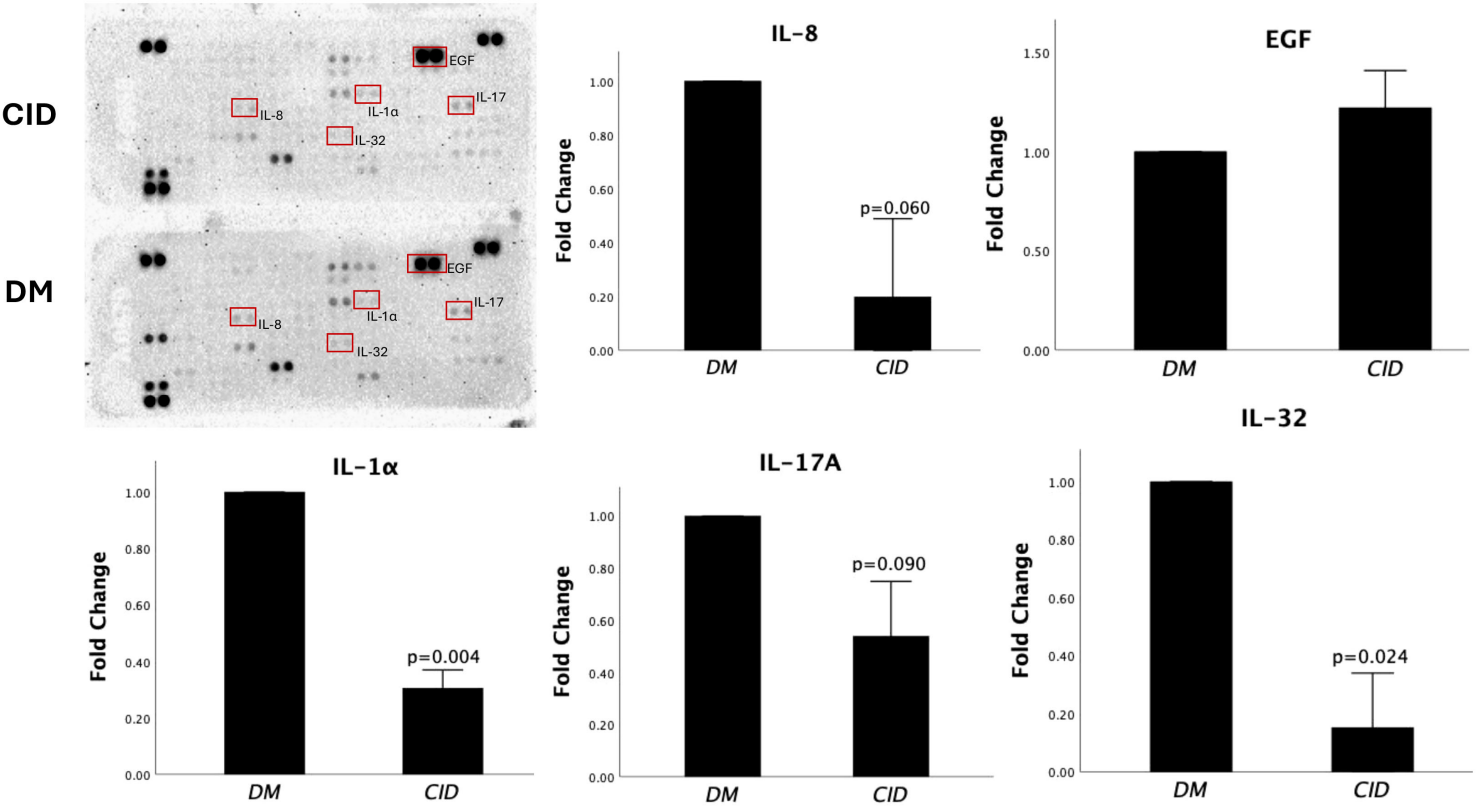
Rab7 Inhibition induces changes in the proteomic profile of ASCs. The upper left image shows the original scan of the protein array membrane. Noticeable reduction in the levels of inflammatory cytokines IL-1α, IL-8, IL-17A, and IL-32 were observed in CID-treated cells, whereas the expression level of EGF was increased. Bars represent the mean fold change in protein expression ± standard deviation (SD) of two experimental replicates in response to CID treatment compared to the control (DM).

The protein array revealed a decrease in the secretion levels of several proteins, including the inflammatory cytokines, IL-1α, IL-8, IL-17A, and IL-32, in CID-treated cells compared to the control group. These findings suggest that Rab7 inhibition may mitigate inflammatory responses in ASCs. Conversely, the treatment of CID resulted in an upregulation of epidermal growth factor (EGF), suggesting a potential enhancement in cellular mechanisms associated with wound healing and tissue regeneration (**Figure 6**).

### 3.7 Rab7 inhibition enhanced the expression of epithelial differentiation markers in ASCs

**Figure 7:**
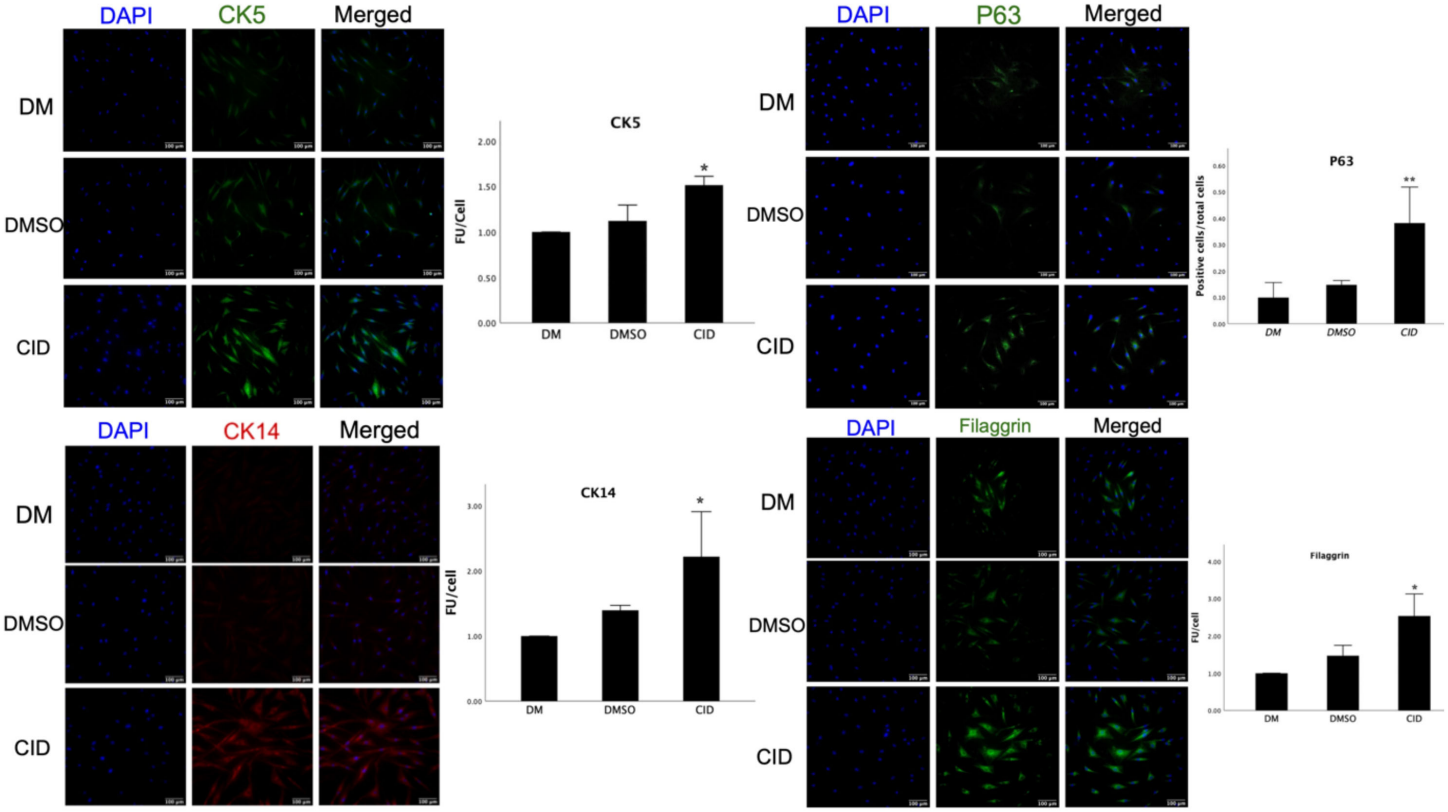
Rab7 Inhibition enhances the expression of epithelial differentiation markers in ASCs. The latter were cultured with differentiation media for ten days with and without CID-1067700, and immunocytochemistry analysis was performed to assess the expression of Cytokeratin 5 and 14, as well as Filaggrin and P63. Nuclei were counterstained with DAPI (blue). The bars indicate the mean fluorescence intensity unit (FU) per cell ± SD of 3 replicates. Asterisks represent significant difference compared to cells treated with differentiation media (DM) only (* p<0.5, ** p<0.01). Scale bars = 100 μm.

Immunofluorescence analysis revealed that Rab7 inhibition significantly increased the expression of several epithelial differentiation markers, including CK5 (p=0.024), CK14 (p=0.023), Filaggrin (p=0.006), and P63 (p=0.003) in ASCs treated with CID-1067700. The upregulation of CK5 and CK14 was validated further, using a conjugated antibody from a different provider (**Supplementary Figure S2**). These results indicate that a 10-day treatment with CID-1067700 not only induced the differentiation of ASCs into basal KLCs but also promoted their differentiation into differentiated KLCs, as supported by the presence of the intermediate differentiation marker, Filaggrin (**Figure 7**).

### 3.8 Rab7 inhibition modulates the mRNA expression of mesenchymal and epithelial markers in ASCs

**Figure 8:**
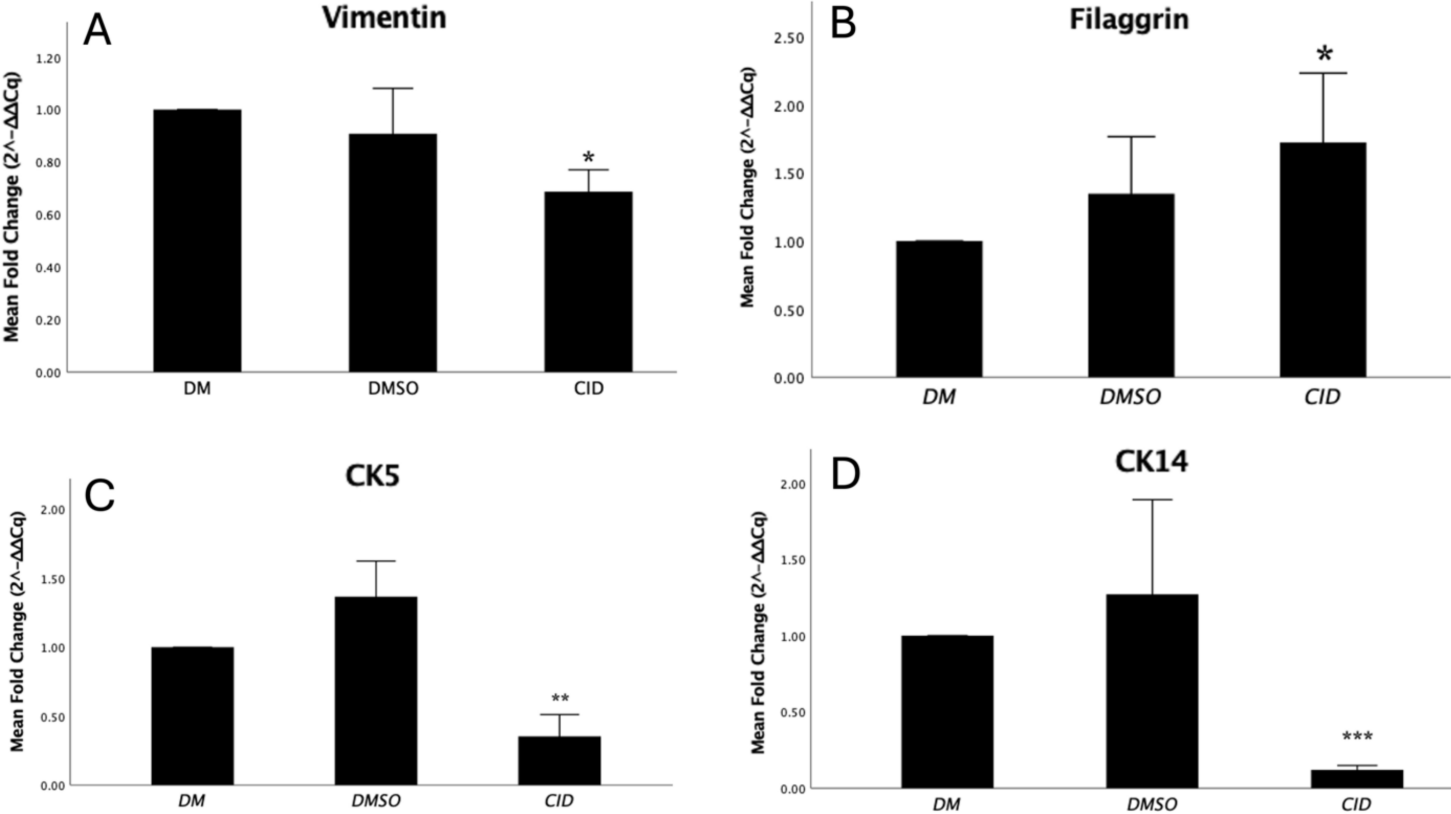
Effect of Rab7 inhibition on the expression of keratinocyte markers in ASCs cells. The gene expression of **(A)** Vimentin, **(B)** Filaggrin, **(C)** Cytokeratin 5, and **(D)** Cytokeratin 14 was measured by Real-time PCR. The expression levels were normalized to *GAPDH* expression and calculated relative to cells treated with differentiation media (DM) alone. Asterisks indicate a significant difference compared to cells treated with DM (* p<0.05, ** p<0.01, *** p<0.001).

The mRNA expression levels of vimentin, an intermediate filament protein predominantly produced by mesenchymal stem cells, as well as the epidermal markers, CK5, CK14, and Filaggrin, were analysed using Real-time PCR. The results indicated that Rab7 inhibition in ASCs led to a significant reduction in vimentin expression (p=0.031) and an increase in Filaggrin expression (p=0.028) as illustrated in **Figures 8A, B**. However, a decrease in the expression of CK5 (p=0.009) and CK14 (p=<0.001) was observed in response to Rab7 inhibition in ASCs cells (**Figures 8C, D**).

## 4 Discussion

The skin has a vast natural regenerative capacity, which is vital to maintain the protectory function against the daily environmental and mechanical challenges. Upon injury, keratinocytes initiate an immunological cascade by secreting a repertoire of cytokines, chemokines, and antimicrobial peptides to stimulate the innate immune response (39, 40). This pro-inflammatory environment facilitates immune cell recruitment and pathogen elimination, which primes the wound bed for subsequent healing processes (41). Moreover, keratinocytes possess remarkable ability to resolve inflammation, fostering the transition from the inflammatory to the proliferative phase of wound healing, and promoting tissue repair (42). However, in the context of chronic wounds, this tightly regulated process is disrupted, leading to prolonged inflammation and tissue degradation. As keratinocytes become arrested in a chronic inflammation state, they exhibit compromised antimicrobial defence capabilities and fail to activate requisite signalling pathways essential for healing progression (6, 43).

Rab GTPases, the largest family of GTPases, are indispensable regulators of membrane trafficking due to their pivotal role in vesicle formation, transport, and fusion in endocytosis, exocytosis, and autophagy (24). Each Ras-related protein (Rab) governs specific steps of vesicular transport, exhibiting precise localization to distinct intracellular compartments. Moreover, several Rab proteins exert control over the degradation and activity of signalling receptors and ligands across developmental signalling pathways (44–46). Rab7 emerges as a key regulator, exerting profound influence over critical developmental signalling pathways. Rab7 regulates the endocytic trafficking of Wnt ligands, receptors, and co-receptors, thereby influencing their stability, availability, and signalling efficacy. Similar effect exerts on the Notch receptors and ligands. Notch is a conserved determinant of cell fate, differentiation, and tissue development (27, 28, 47). This protein plays a role in skin development and regeneration influenced by mitochondrial reactive oxygen species (48). Rab7 extends its regulatory role to the fibroblast growth factor (FGF) signalling pathway, modulating the trafficking and degradation of FGF receptors (FGFRs) and their ligands, thereby dictating the spatial and temporal dynamics of FGF signalling. Rab7 also regulates Hedgehog Signalling Pathway, a critical pathway for embryonic development, tissue patterning, and organogenesis. Furthermore, the impact of Rab7 encompasses melanosome maturation and transport, regulating the trafficking of melanosomes within melanocytes, the specialized pigment-producing cells in the skin (49, 50). These cumulative insights underscore the role of Rab7 in modulating the activation, availability, and efficacy of key developmental signalling pathways. Thus, Rab7 exerts profound influence over cell fate determination, tissue morphogenesis, and organ development. Despite the critical role of membrane trafficking in steering stem cell differentiation, existing differentiation protocols predominantly emphasize targeting signalling pathways and overlook the intracellular vesicle trafficking as a complementary approach for modulating stem cell fate.

In this study, the potential influence of Rab7 in enhancing the differentiation ability of ASCs into KLCs was explored. ASCs were cultured in differentiation media, which has previously been shown to promote the differentiation into KLCs (34). The cells were cultured in the presence or absence of CID-1067700, a competitive Rab7 inhibitor, and the differentiation process was assessed based on the cell morphology and their expression of keratinocyte markers, including Cytokeratin 5 (CK5), Cytokeratin 14 (CK14), P63, and Filaggrin.

Morphological observations revealed that Rab7 inhibition induced a noticeable flattening of ASCs after ten days of treatment. Such morphology suggests that the cells were undergoing the differentiation process, as keratinocytes adopt a flattened shape upon differentiation to provide structural rigidity and mechanical strength, which are key characteristic of the skin epithelium (51). CK5 and CK14 were utilised as early differentiation markers due to their expression in the basal layer of the epidermis. In contrast, filaggrin, is an intermediate marker of epidermal differentiation primarily expressed in well-differentiated keratinized epithelial cells, and it plays a crucial role in regulating epidermal homeostasis (51).

This study revealed that Rab7 inhibitor increased the protein expression of all these markers as early as ten days in culture. However, the mRNA expression of the basal keratinocyte markers CK5 and CK14 was decreased following Rab7 inhibition. This observation suggests that the Rab7 inhibitor effectively promotes the differentiation of adipose-derived stem cells into keratinocyte-like cells. This aligns with the role of Rab7 in regulating membrane trafficking and autophagy, processes crucial for cellular differentiation (37). However, the decrease in mRNA expression of basal keratinocyte markers CK5 and CK14 indicates a complex regulatory mechanism at play. One possible explanation is that Rab7 inhibition may lead to post-transcriptional modifications or stabilization of these proteins, resulting in higher protein levels despite lower mRNA expression. This phenomenon can occur due to enhanced protein stability or reduced degradation, which is often observed in differentiated cells where protein turnover rates are lower (52). Additionally, Rab7’s involvement in autophagy and endocytic pathways might influence the cellular environment, promoting differentiation signals that enhance protein expression while simultaneously downregulating mRNA synthesis as the cells transition through the differentiation state (37). This dual effect underscores the importance of Rab7 in coordinating the balance between stem cell maintenance and differentiation, highlighting its potential as a therapeutic target for enhancing stem cell-based regenerative therapies. Furthermore, we suggest that Rab7 inhibition may facilitate ASC progression through the differentiation process, as basal keratinocytes typically undergo both morphological and biochemical changes during differentiation. These changes include the production of flattened cells and changes in the pattern of keratin expression (53). For example, basal keratinocytes express CK5 and CK14 (54, 55). As they progress into the suprabasal compartment, the transcription of the basal keratins (CK5 and CK14) is downregulated and the transcription of a new set of differentiation state–specific markers, such as filaggrin, is concomitantly induced (54–56). Filaggrin, produced by spinous keratinocytes in the granular layer, facilitates the packing of keratin filaments during terminal differentiation of keratinocytes (57). The upregulation of filaggrin accompanied by the downregulation of CK5 and CK14 gene expression supports this explanation. For further validation, future studies should examine the temporal changes in mRNA expression of CK5 and CK14 in ASCs treated with the Rab7 inhibitor at various stages of differentiation. Time-course experiments would help elucidate whether Rab7 inhibition accelerates the downregulation of basal keratins and the upregulation of terminal differentiation markers.

Microarray analysis revealed substantial changes in gene expression profiles, with 134 differentially expressed genes (DEGs) identified in CID-treated ASCs. Pathway enrichment analysis highlighted the significant impact of Rab7 inhibition on various immune-related pathways. Notably, the chemokine signalling pathway, TNF signalling pathway, cytokine-cytokine receptor interactions, and IL-17 signalling pathway were among the pathways affected by CID treatment. These pathways play critical roles in inflammation and immune response, the two critical processes implicated in the pathogenesis of chronic skin wounds (6). These findings indicates that Rab7 inhibition does not only promote keratinocyte differentiation but may also modulate immune functions.

Prediction analysis using the iPathwayGuide software predicted the euchromatin histone lysine methyltransferase 2 (Ehmt2) as the top activated upstream regulator in CID-treated ASCs. Ehmt2 is known for its role in epigenetic regulation as well as modulating critical biological processes, such as cell cycle regulation, lineage commitment, and differentiation (58). As a repressor, Ehmt2, in conjunction with the Ehmt1/2 complex, serves as a central regulator of nuclear epigenetic processes essential for proper developmental progression from early embryonic stages to terminal differentiation (59–61). Epigenetic regulation is well known to be involved in stem cell differentiation into several lineages (62–65). Ehmt2 has been implicated in the terminal commitment of various lineages, including blood, cardiac, retinal, neural, muscle, and germline lineages (66–72). Moreover, Ehmt2 exert a transcriptional co-activator function, distinct from its classical role in methylation (73). Ehmt2 is also indispensable for the activation of critical genes involved in early development, such as p21 and β-Globin as well as Nfe2 and Runx2 (58, 74). The latter is known to promote the differentiation of ASCs to the epidermal lineage (75). These effects are expected to influence the wound healing process, although we believe that such corelation is understudied. The role of epigenetics in wound healing has been extensively reviewed. The migrating cells at the wound edge were shown to change their epigenetic signature to allow migration and wound closure. The involvement of histone methyltransferases, such as Ezh1 and Ezh2, was crucial for this purpose (76). Furthermore, histone methylation was vital for the determination of tissue macrophage properties as well as peripheral blood mononuclear cells, affecting the wound transition from the inflammatory phase (77). However, the role of Ehmt2 in wound repair needs to be experimentally verified to confirm the potential involvement in the healing process and skin regeneration. Knocking down Ehmt2 in ASC52 cell line can characterize its role in epidermal differentiation in the presence and absence of Rab7 inhibitor.

Furthermore, the protein array data revealed upregulation of EGF in the CID-treated ASCs, indicating a beneficial shift towards enhanced keratinocyte differentiation and wound healing. EGF is a critical regulator of cell proliferation and differentiation, particularly within the epidermis. By promoting keratinocyte migration and proliferation, EGF plays an important role in the re-epithelialization process during wound healing (78, 79). The observed increase in EGF levels in Rab7-inhibited ASCs suggests a potential mechanism for the enhancement of KLCs differentiation and indicates a potential role in accelerating wound healing.

Conversely, the inflammatory cytokines IL-1β and IL-6 were predicted as the top inhibited upstream regulators. This aligns with the observed downregulation of several inflammatory cytokines, including IL-1α, IL-8, IL-17A, and IL-32, in the protein array analysis of the supernatant from CID-treated cell culture. The reduction in these cytokines indicates a potent anti-inflammatory effect of Rab7 inhibition in ASCs, which could be beneficial in managing chronic wounds characterized by persistent inflammation. Recent study by Sonmez Kaplan et al. (80) showed that stimulation with these inflammatory cytokines significantly inhibited the differentiation of dental pulp stem cells into osteogenic, chondrogenic, and adipogenic lineages. Moreover, IL-1β-induced spheres displayed enhanced stemness and drug resistance in colon cancer cells, accompanied by epithelial-mesenchymal transition (80). These findings elucidate the interplay between inflammatory cytokines and stem cell behaviour, highlighting their negative regulatory roles in stem cell differentiation. This represents another advantage for Rab-7 inhibitor as an additive to stem cell differentiation programs and opens a new paradigm for research.

The decrease in pro-inflammatory cytokines observed in this study is of particular interest because of their established roles in wound healing. Elevated levels of IL-17A are commonly associated with chronic wounds. An obvious example is IL-17A rich wound fluid isolated from venous ulcers and lesional tissues of patients with pyoderma gangrenosum (81, 82). Furthermore, IL-17A exhibits synergistic effects with other pro-inflammatory cytokines such as TNF-α, IL-1, and IL-6, exacerbating the inflammatory response and contributing to wound chronicity (83, 84). The dysregulation of chemokine signalling can lead to excessive influx of immune cells into the skin, leading to inflammation and tissue damage (85). Similarly, dysregulated cytokine-cytokine receptor interactions can promote inflammatory responses, immune cell activation, and tissue damage (86). Furthermore, advanced glycation end products (AGEs) and their receptor (RAGE) are implicated in diabetic complications, including diabetic foot ulcers (DFU). Elevated AGE-RAGE levels induce the production of pro-inflammatory cytokines and reactive oxygen species, thereby contributing to the prolonged inflammatory phase and delayed wound healing (87).

Targeting Rab7 holds a great potential for treating chronic skin wounds for several reasons. Firstly, the upregulation of keratinocyte markers indicates that ASCs can differentiate into keratinocytes, which may improve the skin barrier (88). This could lead to the development of topical treatments or cell-based therapies that promote keratinocyte formation and improve skin integrity. Secondly, Rab7 inhibition may attenuate the inflammatory environment in wounds associated with autoimmune diseases through the downregulation of inflammation-related pathways. This could help balance the immune system dysregulation and promote wound healing, both by promoting the natural healing mechanism and adding KLCs as cell-based therapy (6). The clinical translation can also include the production of new anti-inflammatory treatments that specifically target Rab7, potentially reducing the need for systemic immunosuppressive therapies and their associated side effects. Finally, by understanding the specific genes and pathways inhibited by Rab7, a more personalized treatment approach can be developed, tailored to individual patients’ molecular characteristics, which potentially improves treatment responses and outcomes. The insights gained from studying Rab7 could inspire new therapeutic strategies, such as the use of gene editing technologies (e.g., CRISPR) to modulate Rab7 activity in chronic wounds.

However, it’s essential to acknowledge the limitations of our study, including the use of a cell line rather than primary cells. While the ASC52telo cell line offers practical advantages in experimental settings, including reproducibility, scalability, and the absence of donor variability, they may not fully replicate the heterogeneity and physiological context of primary cells. Additionally, these cells could be expected to exhibit potential alterations in gene expression and epigenetic profiles due to the introduction of hTERT during their immortalization process, which may influence their differentiation potential and responses to external stimuli. In fact, ASC52telo cells have been demonstrated to retain key characteristics of primary ASCs, making them a valuable model for controlled mechanistic studies, such as those investigating signalling pathways and differentiation dynamics (21, 31, 33). Prior studies have successfully used these cells to validate platforms for stem cell culture and expansion under defined, Xeno-free, and serum-free conditions, highlighting their suitability for stem cell modelling. These studies also emphasize the compatibility of ASC52telo cells with microcarrier-based systems and their ability to reproduce typical features of primary ASCs, making them a robust model for investigating transcriptional dynamics in controlled environments (e.g., bioreactors), as well as other tissue engineering applications (31). Additionally, ASC52telo cells have been featured in research exploring signalling pathways and cellular differentiation processes, further validating their utility in understanding molecular mechanisms and transcriptional regulation in stem cell biology​ (32). Nonetheless, to enhance the translational relevance of our findings and address potential biases introduced using an immortalized cell line, future studies should integrate primary ASCs to confirm findings and assess inter-individual variability. Incorporating organoid models or *in vivo* approaches would further contextualize these results within a physiologically relevant environment. Investigating the application method in animal models would be of special interest for clinical application. Systemic application versus local application as a solution or in combination with biomaterial, can provide an insight regarding the local effect on the wound as well as the possible side effects. Nevertheless, 2.5 mg/kg of CID were injected intraperitoneally in rats without reported side effects (89). Another limitation of this study is the reliance on immunofluorescence as the sole proteomic evidence of stem cell differentiation into the epithelial lineage. Characterizing differentiation using Western blotting, protein arrays, or other proteomic techniques would provide further confirmation of the differentiation status. Other Rab7 inhibitors, such as VP 3.15., can be evaluated in the future for further validation of the cause-effect. Moreover, functional assays focusing on Rab7 inhibition’s mechanistic effects on stem cell differentiation and inflammation would complement transcriptomic and proteomic analyses, providing a comprehensive understanding of the observed phenomena, including wound healing assay.

## 5 Conclusion

The current study highlights novel regulatory mechanisms governing stem cell differentiation and inflammation in the context of skin regeneration. The identification of Rab7 and Ehmt2 as key regulators of ASCs differentiation opens new avenues for therapeutic intervention, offering potential targets for developing innovative treatments aimed at enhancing tissue repair and regeneration. Further investigation into the potential crosstalk between Rab7, Ehmt2, and inflammatory cytokines holds promise for uncovering novel therapeutic targets and advancing the field of regenerative medicine.

## Data Availability

The datasets presented in this study can be found in online repositories. The names of the repository/repositories and accession number(s) can be found below: https://www.ncbi.nlm.nih.gov/geo/, GSE275489.

## Supplementary material

Supplementary Figure 1 Rab7 inhibition induces transcriptomic changes in ASCs. Heatmap analysis displaying the differentially expressed genes (DEGs) in ASCs following Rab7 inhibition. Samples with relatively high expression of a given gene are shown in red, while those with relatively low expression are shown in blue. Clustering of the samples is illustrated by the dendrogram on top.

Supplementary Figure 2 Immunofluorescence analysis was repeated using conjugated primary antibodies from a different vendor to further confirm the expression of cytokeratin 5 and 14, which are **(A)** CoraLite^®^555 Cytokeratin 5 antibody (1:200, Proteintech, Cat. CL555-28506) and **(B)** CoraLite^®^ Plus 488 Cytokeratin 14 antibody (1:200, Proteintech, Cat. CL488-10143). Data are presented as mean fluorescence intensity unit (FU) per cell ± SD of two replicates, five fields were analysed in each replicate and p < 0.05 was considered significant. Scale bars = 200 μm. The results are consistent with previous findings, confirming the upregulation of CK5 and CK14 in response to CID.

Supplementary Table 1 List of the differentially regulated genes.
