# Supplementary material for: Rab7 inhibitor enhances stem cell differentiation into keratinocyte-like cells with anti-inflammatory properties
Source: Front Immunol. 2025 May 26;16:1503007. doi: 10.3389/fimmu.2025.1503007 (PMC12146274; doi:10.3389/fimmu.2025.1503007)
Supplement: Supplementary Figure 1 — Rab7 inhibition induces transcriptomic changes in ASCs. Heatmap analysis displaying the differentially expressed genes (DEGs) in ASCs following Rab7 inhibition. Samples with relatively high expression of a given gene are shown in red, while those with relatively low expression are shown in blue. Clustering of the samples is illustrated by the dendrogram on top. [file SupplementaryFile1.docx]

**Supplementary Figure 1**


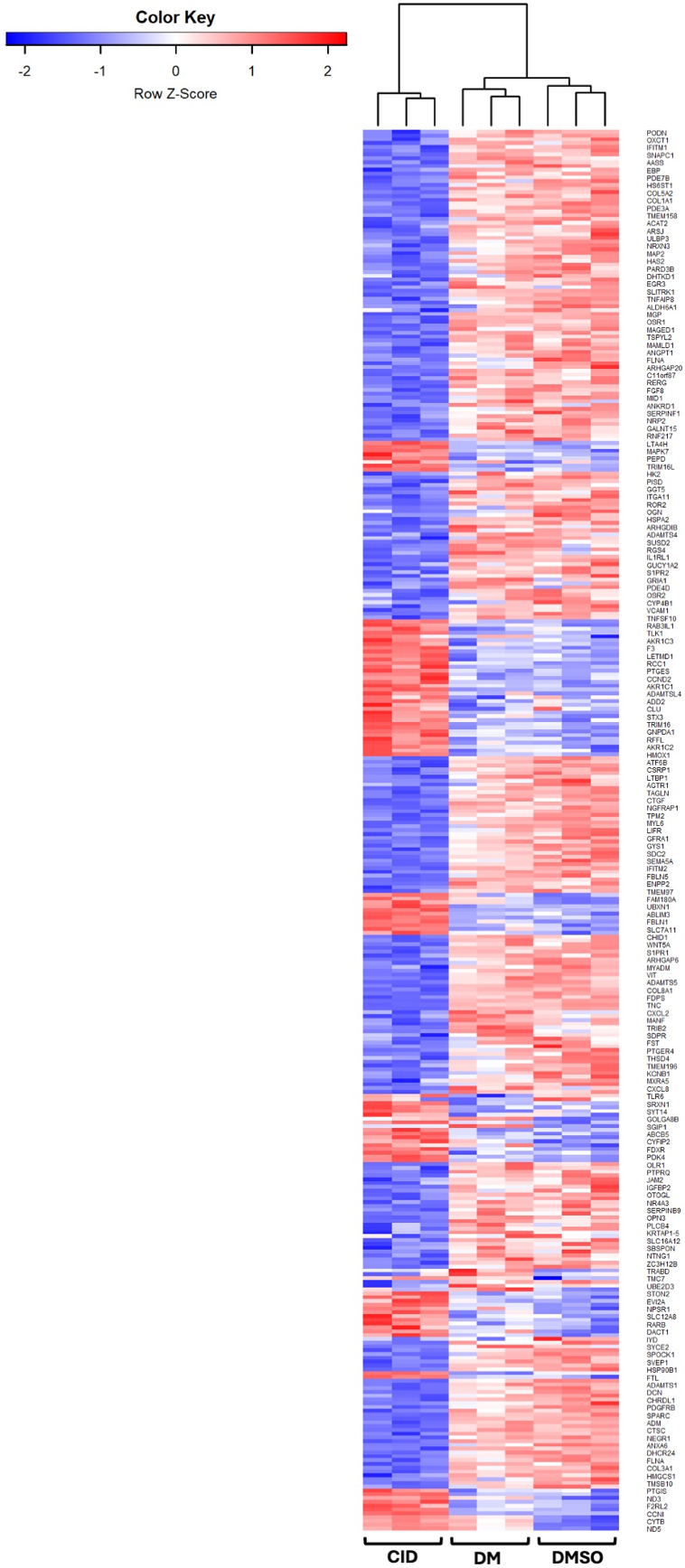


**Figure S1.** Rab7 inhibition induces transcriptomic changes in ASCs. Heatmap analysis displaying the differentially expressed genes (DEGs) in ASCs following Rab7 inhibition. Samples with relatively high expression of a given gene are shown in red, while those with relatively low expression are shown in blue. Clustering of the samples is illustrated by the dendrogram on top.

**Supplementary Figure 2**


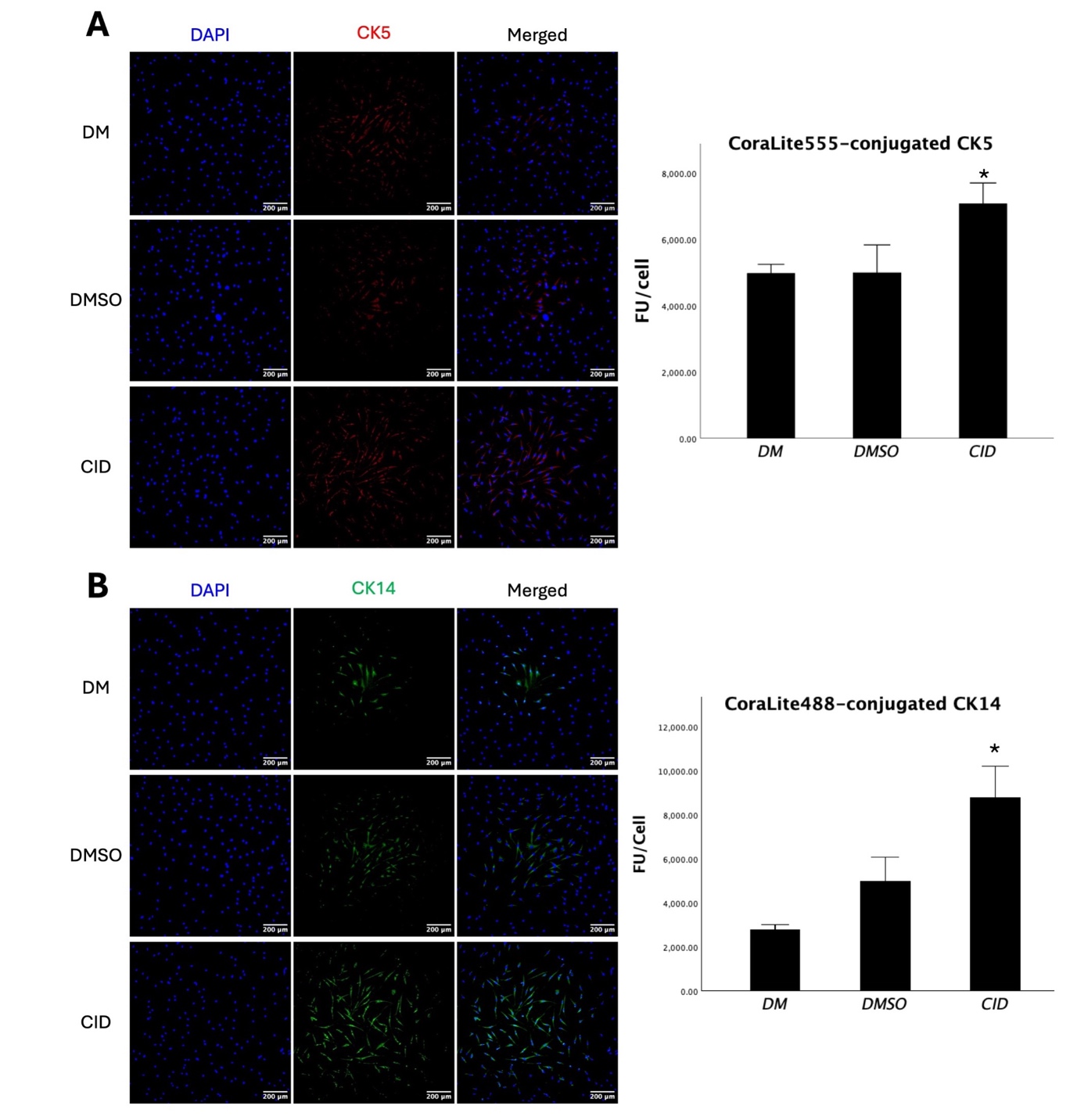


**Figure S2.** Immunofluorescence analysis was repeated using conjugated primary antibodies from a different vendor to further confirm the expression of cytokeratin 5 and 14, which are A) CoraLite®555 Cytokeratin 5 antibody (1:200, Proteintech, Cat. CL555-28506) and B) CoraLite® Plus 488 Cytokeratin 14 antibody (1:200, Proteintech, Cat. CL488-10143). Data are presented as mean fluorescence intensity unit (FU) per cell ± SD of two replicates, five fields were analysed in each replicate and p < 0.05 was considered significant. Scale bars = 200 μm. The results are consistent with previous findings, confirming the upregulation of CK5 and CK14 in response to CID.
