# Supplementary material for: Rab7 inhibitor enhances stem cell differentiation into keratinocyte-like cells with anti-inflammatory properties
Source: Front Immunol. 2025 May 26;16:1503007. doi: 10.3389/fimmu.2025.1503007 (PMC12146274; doi:10.3389/fimmu.2025.1503007)
Supplement: Supplementary Figure 2 — Immunofluorescence analysis was repeated using conjugated primary antibodies from a different vendor to further confirm the expression of cytokeratin 5 and 14, which are (A) CoraLite®555 Cytokeratin 5 antibody (1:200, Proteintech, Cat. CL555-28506) and (B) CoraLite® Plus 488 Cytokeratin 14 antibody (1:200, Proteintech, Cat. CL488-10143). Data are presented as mean fluorescence intensity unit (FU) per cell ± SD of two replicates, five fields were analysed in each replicate and p < 0.05 was considered significant. Scale bars = 200 μm. The results are consistent with previous findings, confirming the upregulation of CK5 and CK14 in response to CID. [file Table1.docx]

**Supplementary Table 1**

Table S1. List of the differentially regulated genes

| Gene | Fold Change | P-val | Gene | Fold Change | P-val | Gene | Fold Change | P-val |
| --- | --- | --- | --- | --- | --- | --- | --- | --- |
| SAA2 | -9,86 | 4,90E-08 | PTPRQ | -2,62 | 4,78E-05 | TMEM158 | -3,65 | 0,0002 |
| SAA1 | -9,11 | 7,53E-08 | ENPP2 | -2,44 | 5,06E-05 | HPD | -2,02 | 0,0002 |
| MGP | -9,74 | 1,40E-07 | AKR1C3 | 2,46 | 5,60E-05 | BHLHE41 | 2,22 | 0,0002 |
| CLEC3B | -7,19 | 1,57E-07 | KCNQ3 | 2,59 | 5,90E-05 | PDE7B | -1,93 | 0,0002 |
| TNC | -7,43 | 1,82E-07 | ITGB8 | -3,89 | 6,23E-05 | CXCL5 | -2,17 | 0,0002 |
| IL1RL1 | -6,44 | 3,39E-07 | APOD | -2,55 | 6,28E-05 | TRIM16 | 2,12 | 0,0002 |
| RERG | -4,85 | 8,54E-07 | MAMLD1 | -2,37 | 6,77E-05 | MFI2 | 1,94 | 0,0002 |
| HMOX1 | 8,28 | 1,30E-06 | CCL2 | -2,26 | 7,30E-05 | CHID1 | -2,16 | 0,0002 |
| SUSD2 | -4,33 | 2,15E-06 | DPP4 | 2,26 | 7,83E-05 | IGFBP7 | -1,96 | 0,0002 |
| XPNPEP2 | -5,24 | 2,66E-06 | ZC3H12B | -2,6 | 8,03E-05 | PRODH | -2,16 | 0,0002 |
| COL8A1 | -3,27 | 4,44E-06 | LBH | -2,6 | 8,12E-05 | FAM153B | -2,86 | 0,0002 |
| ROR2 | -3,32 | 6,01E-06 | OSR2 | -2,28 | 8,16E-05 | IFITM3 | -2,08 | 0,0002 |
| ZNF385D | -3 | 7,67E-06 | GFRA1 | -2,14 | 8,54E-05 | IGFBP2 | -2,8 | 0,0002 |
| TFPI2 | -3,72 | 7,73E-06 | EGR3 | -2,13 | 8,59E-05 | XRCC2 | -1,95 | 0,0002 |
| VCAM1 | -6,55 | 8,19E-06 | PDE1A | -3,88 | 8,64E-05 | SDPR | -2 | 0,0002 |
| CRLF1 | -2,84 | 9,68E-06 | TRIB2 | -2,23 | 8,92E-05 | LOC105377348 | -1,96 | 0,0002 |
| ROS1 | -3,02 | 1,02E-05 | MOK | -2,17 | 9,32E-05 | OPN3 | -2,58 | 0,0002 |
| CDK15 | 2,98 | 1,05E-05 | ENC1 | -3,39 | 0,0001 | PILRA | -2,15 | 0,0002 |
| SCN7A | -4,15 | 1,06E-05 | BDKRB2 | 2,48 | 0,0001 | S1PR1 | -2,18 | 0,0002 |
| VIT | -2,84 | 1,18E-05 | HSPB7 | 2,63 | 0,0001 | GPAT3 | 2,13 | 0,0002 |
| RDH10 | -3,66 | 1,22E-05 | PLCB4 | -2,2 | 0,0001 | MAOA | -3,03 | 0,0002 |
| OTOGL | -7,53 | 1,31E-05 | AKR1C2 | 2,33 | 0,0001 | ACAT2 | -2,39 | 0,0002 |
| PLP1 | -3,2 | 1,36E-05 | TMEM45A | -2,24 | 0,0001 | PLEKHG4 | -2,26 | 0,0002 |
| CXCL6 | -2,94 | 1,38E-05 | MAP2 | -2,34 | 0,0001 | PDK4 | 3,27 | 0,0002 |
| CXCL1 | -4,12 | 1,50E-05 | ENTPD1 | -3,29 | 0,0001 | LURAP1L | -1,88 | 0,0002 |
| SLITRK1 | -2,76 | 1,67E-05 | F3 | 2,05 | 0,0001 | SERPINB9 | -1,91 | 0,0003 |
| NRG1 | -3,21 | 1,72E-05 | PRKD1 | -2,11 | 0,0001 | GLIS3 | -1,94 | 0,0003 |
| MATN2 | 2,64 | 2,01E-05 | COL3A1 | -2,12 | 0,0001 | COL1A1 | -2,25 | 0,0003 |
| GLIPR1 | -2,64 | 2,44E-05 | NR4A3 | -2,47 | 0,0001 | ADAMTS9 | -2,58 | 0,0003 |
| CPM | -2,54 | 2,45E-05 | TAGLN | -2,1 | 0,0001 | ADD2 | 1,9 | 0,0003 |
| FGF7 | -2,49 | 2,45E-05 | DNAJC6 | -2,04 | 0,0001 | PCDH7 | -2,08 | 0,0003 |
| HAS2 | -2,46 | 2,73E-05 | GGT5 | -2,09 | 0,0001 | MVD | -2,13 | 0,0003 |
| RGS4 | -2,6 | 2,90E-05 | JAG1 | -2,68 | 0,0001 | TAS2R13 | 1,91 | 0,0003 |
| FDPS | -2,54 | 3,05E-05 | MAMDC2 | -2,21 | 0,0001 | PTGIS | 2,33 | 0,0003 |
| SPARCL1 | -2,87 | 3,61E-05 | GRIA1 | -2,12 | 0,0001 | TNFRSF11B | -1,9 | 0,0003 |
| PLIN2 | 2,47 | 3,80E-05 | NEGR1 | -2,13 | 0,0001 | GUCY1A2 | -3,3 | 0,0003 |
| PRG4 | -3,84 | 3,89E-05 | PDE4D | -2,2 | 0,0001 | PIK3R3 | -2,3 | 0,0003 |
| PID1 | -2,39 | 3,94E-05 | ARHGDIB | -2,12 | 0,0001 | OSGIN1 | 2,04 | 0,0003 |
| TRIM16L | 2,31 | 3,98E-05 | AKR1C1 | 2,72 | 0,0001 | TMEM196 | -2,04 | 0,0003 |
| PLXNA4 | -2,8 | 4,22E-05 | ZNF385D | 2,02 | 0,0002 | ADAMTS1 | -1,85 | 0,0003 |
| ABCC2 | -2,38 | 4,29E-05 | ARL4C | 2,17 | 0,0002 | GRAMD4 | 1,99 | 0,0003 |
| C11orf87 | -3,06 | 4,29E-05 | TNFSF10 | -3,54 | 0,0002 | WNT5A | -1,99 | 0,0003 |
| ADM | -2,35 | 4,40E-05 | C8orf34 | -2,12 | 0,0002 | SLC12A8 | 2,25 | 0,0003 |
| CXCL8 | -2,59 | 4,55E-05 | MANF | -2,21 | 0,0002 | TLR6 | 2,1 | 0,0003 |
| PLPPR4 | -2,29 | 4,78E-05 | PDE3A | -2,25 | 0,0002 |  |  |  |
